# Trophic State and Toxic Cyanobacteria Density in Optimization Modeling of Multi-Reservoir Water Resource Systems

**DOI:** 10.3390/toxins6041366

**Published:** 2014-04-22

**Authors:** Andrea Sulis, Paola Buscarinu, Oriana Soru, Giovanni M. Sechi

**Affiliations:** 1 Hydraulic Sector, Department of Civil an Environmental Engineering and Architecture, University of Cagliari, via Marengo 2, Cagliari 09123, Italy; E-Mail: sechi@unica.it; 2 Sardinian Water System Management Authority, Regional Government, via Mameli 88, Cagliari 09123, Italy; E-Mails: paola.buscarinu@enas.sardegna.it (P.B.); orn.soru@gmail.com (O.S.)

**Keywords:** trophic state index (*TSI*), Cyanobacteria, harmful algal blooms (HAB), optimization modeling, Flumendosa-Campidano water system

## Abstract

The definition of a synthetic index for classifying the quality of water bodies is a key aspect in integrated planning and management of water resource systems. In previous works [1,2], a water system optimization modeling approach that requires a single quality index for stored water in reservoirs has been applied to a complex multi-reservoir system. Considering the same modeling field, this paper presents an improved quality index estimated both on the basis of the overall trophic state of the water body and on the basis of the density values of the most potentially toxic Cyanobacteria. The implementation of the index into the optimization model makes it possible to reproduce the conditions limiting water use due to excessive nutrient enrichment in the water body and to the health hazard linked to toxic blooms. The analysis of an extended limnological database (1996–2012) in four reservoirs of the Flumendosa-Campidano system (Sardinia, Italy) provides useful insights into the strengths and limitations of the proposed synthetic index.

## 1. Introduction

Integrated Water Resources Management (IWRM) requires the analysis of quantitative, as well as qualitative aspects in a common strategy. Over the past few decades, a number of simulation and optimization models have been developed to manage multi-reservoir systems [3]. Mathematical optimization procedures can be constructed to efficiently solve an optimization model that adequately approximates the real water system management requirements, and a subsequent simulation phase can greatly narrow down the search for the optimum [4,5,6,7]. When considering the tools needed for adequately approaching mathematical optimization to real problems, water quality characterization requirements are critical components in constructing the optimization model. Optimization models defining water quality policy can be developed at different levels of complexity [8], from conceptual models to physically-based models, the latter often suffering from limited computational feasibility and great computational complexity.

In the Mediterranean region, eutrophication is one of the most serious problems affecting the quality of water stored in surface reservoirs. The increase in nutrients leads to the greater productivity of the water system, which may result in an excessive increase in algal biomass or other primary producers, such as macrophytes. Excessive algal biomass can seriously affect water quality, especially if it creates anaerobic conditions. A trophic state is a multidimensional concept, involving different aspects; nevertheless, single criterion alternatives to multi-parameter indices have also been developed. Several researchers have proposed relationships between algal biomass, nutrient concentration and nutrient load. Carlson [9] suggested that algal biomass is the key indicator for trophic state classification, and chlorophyll-a (Chl-a) is the best for estimating algal biomass in most reservoirs. The classification of the quality of water held in reservoirs based on the trophic state index (*TSI*) proposed by Carlson [9] can be inserted into an optimization model to make preliminary assessment of quality related to overall algal biomass concentrations in the period of maximum stratification. *TSI* can be easily used to insert management constraints on water use possibilities, to predict short-term trophic evolution in different climate scenarios and to check the relationships between parameters [2]. This approach can be adopted in simplified models for completely mixed lakes without stratification when considering complex multi-reservoir systems. Nevertheless, the use of *TSI* alone does not provide data concerning algal composition nor does it allow us to establish whether the algal blooms will be made up of a specific type of algae. Some families of microscopic phytoplanktonic algae produce toxins during water body eutrophication phenomena. Particularly in artificial reservoirs, toxin-producing phytoplankton belongs almost exclusively to Cyanobacteria. Toxic blooms of Cyanobacteria are a growing problem throughout the world. Monitoring of the toxicity of these blooms showed that almost half are indeed toxic. The most frequently found toxins, microcystins, are new, oncogenic-risk substances that cannot be eliminated by standard water purification processes. Special filters or costly treatments are needed to prevent them from entering the supply network. In such cases, limitations on resource use based solely on *TSI* values may not be sufficient. This article aims to present a combined *TSI* and algal classification system that could be used to provide a single parameter for classifying reservoir water quality in an optimization model framework treating complex water systems.

The paper is structured as follows: Section 2 briefly presents the different approaches from the literature on the identification of interrelations between different parameters to characterize the trophic state of lakes and reservoir; Section 3 focuses on the phenomena of harmful algal blooms (HAB); Section 4 describes the limnological survey and the evaluation of water quality in the period of 1996–2012 in four main reservoirs in the Flumendosa-Campidano water supply system (Sardinia, Italy); the water quality evaluation (*QE*) is based on the validation of Carlson’s approach [9] and the analysis of the density of algal species specifically considering the class of Cyanobacteria; finally, a synthetic quality index is proposed and evaluated to be inserted in the Water Resources Graphical Interface Decision Support System (WARGI-DSS) [10] in a predictive screening-level model. Some conclusions are drawn to synthesize the major results of the overall analysis and its limitations, and the need for further research in the field of water quality optimization modeling is noted.

## 2. Reservoir Trophic State Characterization

The trophic status in reservoirs is a multidimensional concept involving a wide range of data types (morphological, physical, chemical and biological) that are most notably related to human activity in the basins. To estimate future trends of the eutrophication rate under changing conditions, several models and statistical analyses use biomass, an indirect measure of which is given by Chl-a concentration. Several researchers [9,11,12,13] have found a log-linear relationship between Chl-a and nutrients (nitrogen and phosphorus). The input-output models developed by Dillon and Rigler [14] and by Vollenweider [15] can be used to predict phytoplankton biomass (expressed as Chl-a). Smith [13] developed a theoretical framework that reduces the error of chlorophyll prediction in the Dillon-Rigler model by taking into account the influence of the total phosphorus (TP)/total nitrogen (TN) weight ratio. Smith [13] showed that TN concentration influences chlorophyll concentration, even in lakes where phosphorus alone had previously been presumed to be a limiting factor. In these simple input-output models, other factors that can also influence the trophic status, such as biological interaction, internal nutrient loading and physical conditions, are not assessed. Although these models are only applicable in discrete periods of the year under the very restrictive assumption that a reservoir is a mixed system, these approaches are the most common in the literature.

When analyzing complex phenomena, understanding how these factors are related and inserting them into a model using analytical relations requires great effort. Brylinsky and Mann [16] analyzed the interactions between a large number of variables using a combination of hierarchical models and statistical analyses. Hierarchical models provided a framework for the identification of the relationships between these variables that were tested using correlation and factor analysis. The variables were grouped into those influencing primary production through nutrient availability (depending on the nutrient input, characteristics and area of the drainage basin, the nutrient dilution, precipitation, evaporation, surface area and mean depth and the nutrient distribution, wind, surface area and temperature range of water) and through energy availability (depending on the total incident radiation, reflection and turbidity). They used simple or multiple regression analysis for the comprehension of the type and degree of the relationship between morphological, chemical, physical and biological variables. The results of regression analysis showed that phytoplankton biomass and Chl-a are closely correlated with each other and with phytoplankton production. Multiple regression using biological indices to estimate phytoplankton production also showed that Chl-a and TP explain 45% of phytoplankton production variance.

The various attempts to analyze these complex phenomena in a simplified manner enable a description of the trophic condition of the reservoir using a mathematical modeling approach for complex water supply system management optimization.

To define the water quality index in complex multi-reservoir systems, we use the *TSI* proposed by Carlson [9], which in recent years seems to have gained general acceptance from the limnological community for characterizing a reservoir’s trophic state. *TSI* is evaluated using Chl-a, TP and Secchi disk (SD) transparency measurements. The *TSI* scale shown in Table 1 ranges from zero (ultra-oligotrophic) to 100 (hyper-eutrophic) [17]. High and/or increasing trophic status values indicate an increase in eutrophic conditions (higher biomass).

In accordance with recent legislation in European countries, and specifically in Italy [18], the environmental state is based on four macro descriptors (Chl-a; transparency; total phosphorous; hypolimnion oxygen) and summarized in a *QE* index of five possible numerical values:

*QE* = 1 → excellent;
*QE* = 2 → good;
*QE* = 3 → acceptable;
*QE* = 4 → poor;
*QE* = 5 → bad.

Combining *QE* and *TSI*, the proposed water quality classification for reservoirs is presented in Table 1.

**Table 1:** Relations between trophic state index (*TSI*) values, lake attributes and quality evaluation (*QE*) values.

*QE*	*TSI*	Attributes
1	<40	Oligotrophy
2	40–50	Mesotrophy
3	50–70	Eutrophy
4	70–80	Hypereutrophy
5	>80	Over-hypereutrophy

## 3. Problems Associated with the Presence of Phytoplankton in Water Stored in Reservoirs

In internal low hydro-dynamism water bodies (such as regulation reservoirs with a significant ratio between storage capacity and mean annual inflow), the microscopic organisms making up phytoplankton play a leading role in the primary production of living organic substance or biomass.

While phytoplankton plays a key role in lake ecosystems, its excessive proliferation becomes a serious problem for water stored in reservoirs used for potable and/or recreational supply. The immediate consequences of this excess growth are numerous, ranging from the simple abundance of suspended particles (phytoplankton itself, zooplankton, bacteria, fungi and detritus) to increased concentrations of ammonia, nitrites, hydrogen sulfide, methane, ethane and humic acids, to bad flavor and smell in fish and water, due to the presence of particular algae, and the possible development of toxic algae.

At times, the phenomenon is so obvious, that the naked eye can see the mass of microscopic algae, which produce blooms giving a particular coloring to the water body. The term “algal blooms” indicates a situation in which 80%–90% of the mass of microscopic algae consists of one or two species. In particular, Cyanobacteria may be considered as blooming when their cell number exceeds one million per liter. This coating of microscopic algae covers the surface of the water and decreases its transparency. This, in turn, prevents the penetration of sunlight, something that, coupled with the thermal stratification typical of reservoirs in the Mediterranean area, inevitably engenders conditions of anoxia and, hence, the above-mentioned consequences.

A further alarming aspect associated with eutrophication is the fact that algal species responsible for blooms belong to the taxonomic group of Cyanobacteria, present in surface waters all round the world. Cyanobacteria produce a wide range of toxins, which, according to their effects, are classified as hepatotoxins, neurotoxins, skin irritants and other toxins (Table 2) [19].

**Table 2:** Toxins and producer organisms.

Name		Producer
Neurotoxins	Anatoxin-a	*Anabaena, Aphanizomenon*
	Homo-anatoxin-a	*Oscillatoria (Planktothrix)*
	Anatoxin-a(s)	*Anabaena, Oscillatoria (Planktothrix)*
	Paralytic shellfish poisons (saxitoxins)	*Anabaena, Aphanizomenon*
Hepatotoxins	Cylindrospermopsin	*Aphanizomenon, Cylindrospermopsis*
	Microcystins	*Anabaena, Aphanocapsa, Microcystis, Oscillatoria (Planktothrix)*
	Nodularins	*Nodularia*
Contact irritant-dermal toxins	Aplysiatoxin	*Schizothrix*

Cyanotoxins are produced and contained in the cells during their growth and released into the water mainly during cell lysis (e.g., for senescence or death) rather than by continuous excretion [20]. Thus, a water body populated by toxic species will inevitably become enriched with their toxins, which can affect human beings or animals using the water. The literature reports frequent cases of deadly poisoning of many animal species, both wild and domestic, following consumption of water with potentially toxic Cyanobacteria blooms, the poisoning being due to neurotoxins or hepatotoxins.

Neurotoxins are produced by species, such as *Dolichospermum flos-aquae*, *Dolichospermum spiroides*, *Anabaena circinalis* and *Aphanizomenon flos-aquae*, and genera, such as *Oscillatoria*, *Gomphosphaeria* and *Trichodesmium*. These organisms can, at the same time, produce hepatotoxins, but the latter act more slowly; therefore, the clinical picture is dominated by neurotoxins.

Hepatotoxins are more frequent than neurotoxins and are produced by Cyanobacteria belonging to a number of genera, the most common being *Microcystis*, *Anabaena*, *Oscillatoria* (*Planktothrix*), *Nostoc*, *Nodularia*, *Aphanizomenon* [21,22] and *Cylindrospermopsis*. Among hepatotoxins, the most abundant are microcystins, 50 types of which are known. Their effects on human beings and animals may be summarized as:

- Acute hepatotoxicosis, due to direct ingestion;
- Carcinogenic effect, if ingested in sub-acute doses over a long period of time (hepatic cancer);
- Acute pneumonias, if breathed in.

In Italy, at the present time, microcystins each year affect the reservoirs of seven regions out of twenty. While the World Health Organization has addressed the problem of Cyanobacteria blooms, neither the European Union (EU) nor Italian legislation have established concentration limits for cyanotoxins in water for human consumption and/or recreational use. On the assumption of a three-stage water purification system [2]. In this study, we adopted a threshold value of Cyanobacteria density for potable use (and for cautionary purposes, also for irrigation use) of 100 × 10^6^ cell/L.

## 4. Limnological Survey and Water *QE* in the Flumendosa-Campidano Reservoirs

The Flumendosa-Campidano water supply system extends over southeastern Sardinia (Italy), reaching to the center of the Island. The hydrology is typically Mediterranean, with the alternation of several droughty years with years marked by intense rainfall. Besides being the most extensive on the island, this water system is also the most complex, since it interconnects with other systems, and it is a multi-reservoir and multi-use system. Its pivot is a series of reservoirs, linked in a cascading sequence, whence depart pressure pipelines for residential use and open channels supplying irrigation water to the Campidano plain. An extended description of the main characteristics of the system and the connection scheme made using the graphical interface of WARGI-DSS are reported in Sechi and Sulis [10].

Since the early 1990s, the Sardinian Water System Management Authority (Ente Acque della Sardegna, ENAS) has conducted an intensive monitoring program to identify the water quality status in the most important reservoirs in the system, particularly the following four reservoirs, whose main characteristics are shown in Table 3: (1) Flumendosa at Nuraghe Arrubiu (latitude = 39°42′38′′ N; longitude = 9°17′25′′ E); (2) Mulargia at Monte su Rei (latitude = 39°37′30′′ N; longitude = 9°14′23′′ E); (3) Is Barrocus (latitude = 39°45′10′′ N; longitude = 9°5′32′′ E); and (4) Cixerri at Genna Is Abis (latitude = 39°16′53′′ N; longitude = 8°52′56′′ E).

**Table 3:** The main characteristics of the reservoirs in the Flumendosa-Campidano system.

Reservoir features	Cixerri	Mulargia	Flumendosa	Is Barrocus
Total catchment basin (km^2^)	426.00	1183.16	1004.51	93.00
Reservoir surface at maximum level (km^2^)	4.90	12.40	9.00	6.30
Elevation at maximum regulation level (m)	39.00	258.00	267.00	413.00
Volume at maximum regulation level (×10^6^ m^3^)	23.90	320.70	292.90	11.96

Measurements of the main chemical and biological parameters have been performed by the ENAS since the appearance of algae blooms in reservoirs in the early 1990s. These data seem to suggest that eutrophication and high mineral nutrient load in inflowing water are indeed the major water quality problems of the system. In general terms, only the two larger reservoirs in the system, Flumendosa and Mulargia, have a trophic level variable between oligotrophy and mesotrophy [23]. The other two lakes, Cixerri and Is Barrocus, are in a state of more or less advanced eutrophication, at times hovering between eutrophy and hypertrophy [24]. ENAS measured sampling parameters selected to represent the trophic conditions of the reservoirs, particularly Chl-a, TP and SD transparency. Measurements were taken at least once a month from January 1996 to December 2012, at different depths in all reservoirs.

**Figure 1:**
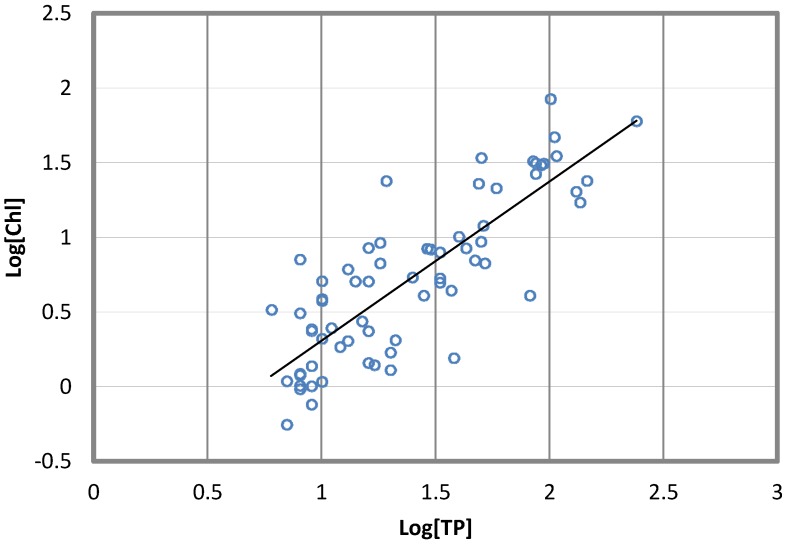
Summer average chlorophyll-a (Chl-a) concentration *vs.* total phosphorus (TP) concentration at the spring overturn in four reservoirs.

Following several studies [11,12,13,14], we used Chl-a as a simple and useful estimator of phytoplankton biomass and found, for the reservoirs in Table 3, a relationship between the average concentration of Chl-a in the summer by using measures of TP during the spring (Figure 1):

log_10_[*Chl*] = 1.065log_10_*TP* − 0.758 (1)

We found that Chl-a in the four reservoirs of the system was quite accurately related to phosphorus concentration (*R* = 0.83). This is a significant factor in water quality management, because it makes it possible to predict the trophic state of reservoirs once the Chl-a concentration is known.

**Figure 2:**
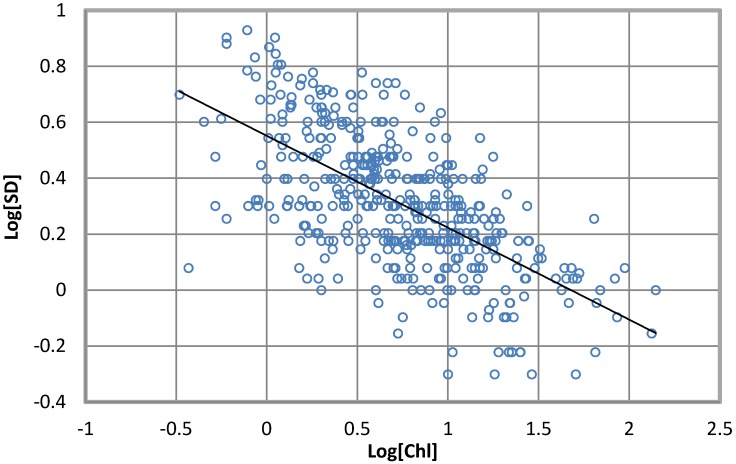
Secchi disk (SD) transparency *vs.* Chl-a concentration near the surface in four reservoirs.

A log-log transformation of the SD transparency and Chl-a concentration data (Figure 2) was used to define the resulting log-linear combination (*R* = 0.64):

log_10_[*SD*] = 0.551 − 0.328 log_10_[*Chl*] (2)

where the SD transparency is in meters and the Chl-a concentration is in milligrams per cubic meter taken near the surface. One possible explanation for the exponential form of Equation (2) is given by Steele [25].

We defined the *TSI* equations specifically suited for the Flumendosa-Campidano water system based on the trophic state definition of the Carlson’s approach [9]:

*TSI* = 10 × (6 − log_2_*SD*) (3)

Combining Equation (3) with the regression of Chl-a against TP (Equation (1)) and of SD against Chl-a (Equation (2)), site-specific equations for *TSI* in the Flumendosa-Campidano water system were assessed. Specifically, *TSI* can be computed from SD transparency, Chl-a concentration and TP using the equations in the forms:

*TSI*(*SD*) = 60 − 14.41ln *SD* (4)

*TSI*(*Chl*) = 2.05 ln *Chl* + 52 (5)

*TSI*(*TP*) = 2.19 ln *TP* + 48.47 (6)

As previously explained, the *TSI* based on Chl, TP and SD can be obtained for the classification of reservoirs. Using the previous equations (Equations (4)−(6)), *TSI*s have been calculated from the observed data, and Table 4 reports the statistical values.

**Table 4:** Statistical summaries of the *TSI* data evaluated from the samples.

Lake	Water quality parameter	Number of samples	Max.	Min.	Mean	Standard deviation
Flumendosa	*TSI* (Chl)	146	30	58	45	4.4
	*TSI* (TP)	145	55	33	44	3.2
	*TSI* (SD)	146	65	26	41	6.8
Cixerri	*TSI* (Chl)	229	69	36	56	5.8
	*TSI* (TP)	249	64	46	55	2.6
	*TSI* (SD)	229	87	38	65	7.6
Is Barrocus	*TSI* (Chl)	249	65	37	51	4.8
	*TSI* (TP)	250	43	59	50	3.1
	*TSI* (SD)	252	70	36	54	6.8
Mulargia	*TSI* (Chl)	239	62	36	48	4.5
	*TSI* (TP)	237	56	37	46	3.1
	*TSI* (SD)	237	73	30	47	9.2

As regards phytoplankton composition, the microscopic observations were carried out on fresh and Lugol fixed samples in 10-mL sedimentation chambers, using an inverted microscope (Axiovert 100, Carl Zeiss Microscopy, LLC, Thornwood, NY, USA). The cell density was determined according to Utermöhl [26] and Lund *et al.* [27]. Species were determined according to Anagnostidis and Komárek [28], Komárek and Anagnostidis [29,30], Huber-Pestalozzi [31], Germain [32], and Cronberg and Annadotter [33]. Species in the four reservoirs mostly belong to five classes: Cyanobacteria, Chlorophyceae, Bacillariophyceae (Diatoms), Cryptophyceae and Conjugatophyceae. Furthermore, Dinophyceae and Chrysophyceae were recorded, but not analyzed, in this paper, because of their low densities. To assess the contribution of each class to total density, each reservoir density value, collected between 1996 and 2012, was processed as follows:

- Mean annual density values (cells/liter) were calculated for each class (absolute and percent value);
- Minimum and maximum values reached by each class in the period under study (1996–2012) were recorded;
- The most representative species in each class were identified.

**Figure 3:**
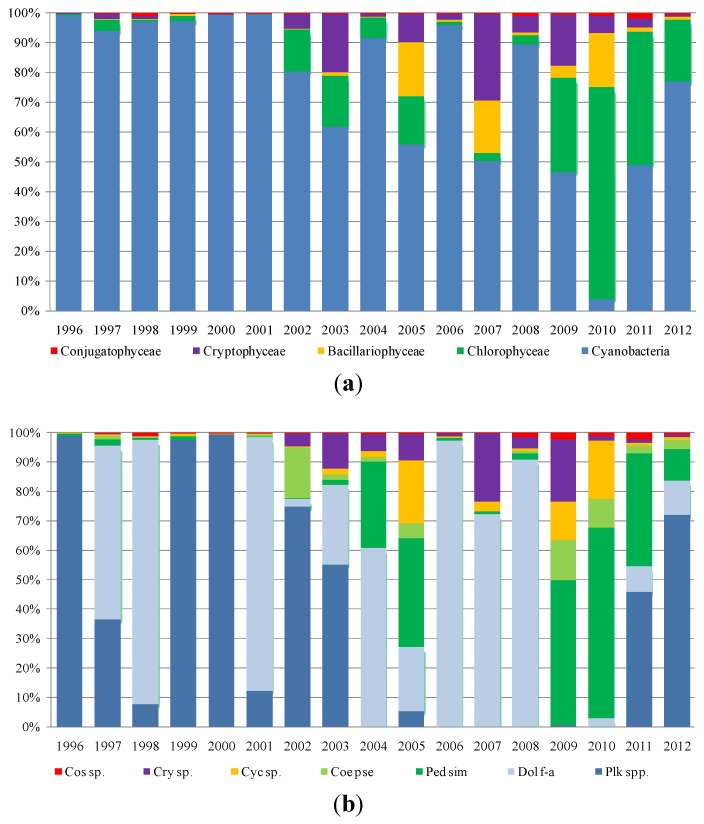
The densities (percentage of cells per liter) of (**a**) classes and (**b**) species in Cixerri reservoir.

**Figure 4:**
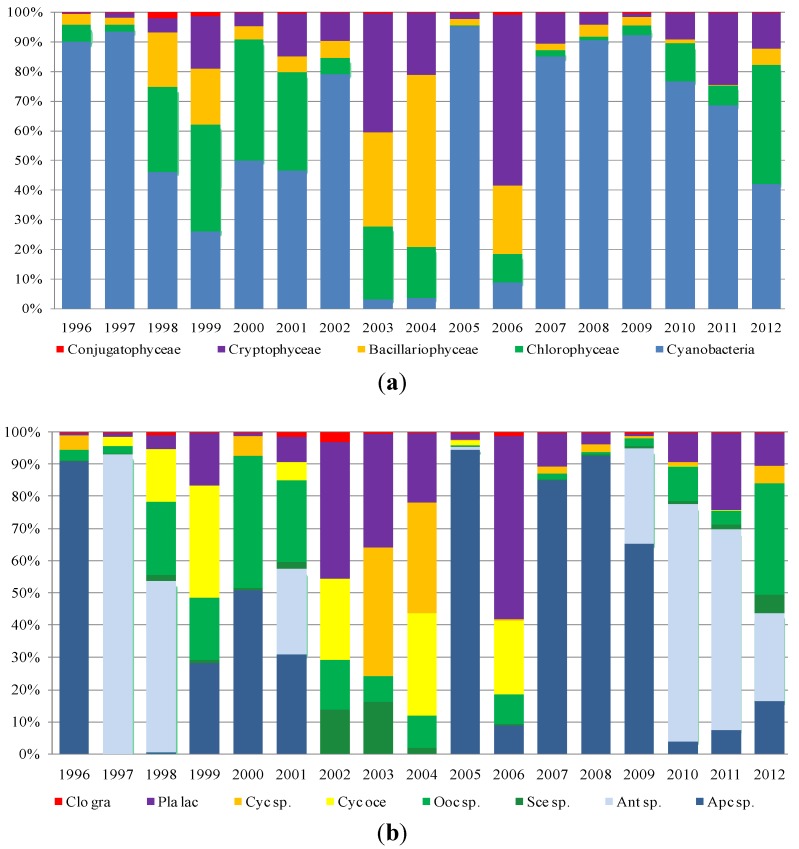
The densities (percentage of cells per liter) of (**a**) classes and (**b**) species in Is Barrocus reservoir.

**Figure 5:**
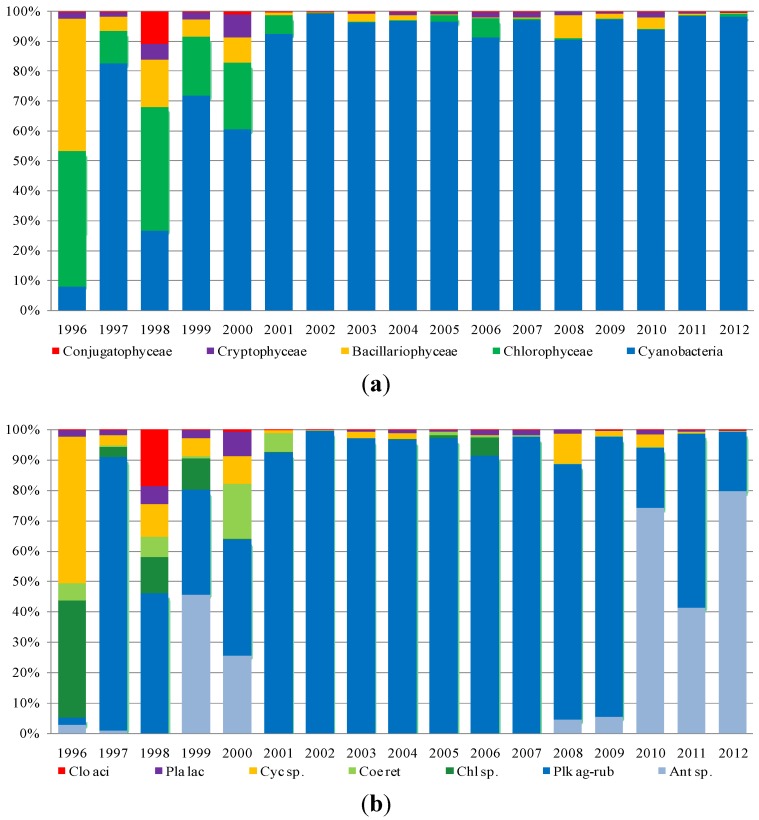
The densities (percentage of cells per liter) of (**a**) classes and (**b**) species in Flumendosa reservoir.

**Figure 6:**
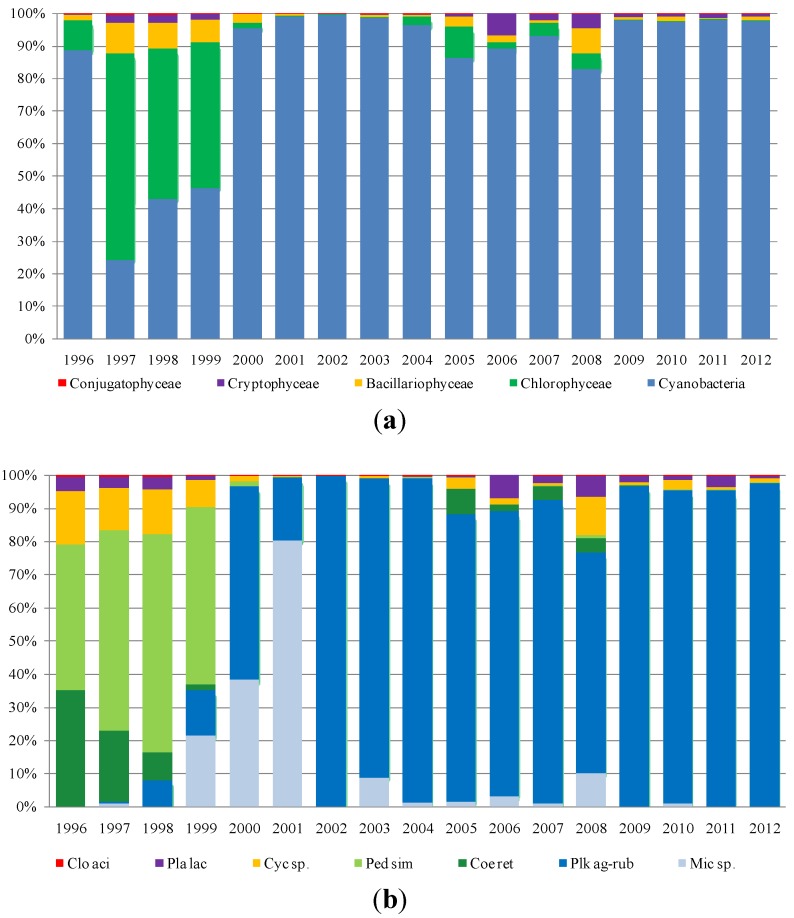
The densities (percentage of cells per liter) of (**a**) classes and (**b**) species in Mulargia reservoir.

Figure 3, Figure 4, Figure 5 and Figure 6 show the data processing output. The graphic depiction enables us to gain an idea of how the density of each class changed over time in each reservoir.

In the Cixerri reservoir (Figure 3), the dominance of Cyanobacteria has been particularly massive in the period between 1996 and 2002, with a maximum peak density value in 2000 equal to 99%, corresponding to a density of 736.051 (×10^3^ cell/L). The *Aphanizomenon* genus (Nostocales order) presented dominance with the species *A. flos-aquae*, *A. gracile* and *A. issatschenkoi*. Some of these blooms have passed one million cells per liter [34]. Negative toxicity assays (enzyme-linked immunosorbent assay (ELISA) kit for saxitoxin) were obtained for these species. The interesting presence should be noticed, in September 2012, of the tropical genus, *Cylindrospermopsis*, which was tested using the ELISA kit for cilyndrospermopsin for the potential toxicity of some of its strains. All the assays were negative. Within the *Dolichospermum* genus, *D. flos-aquae* have dominated more frequently than *D. spiroides* and *D. planctonicum*. Within Oscillatoriales, the *Planktothrix* genus showed a variable concentration with high values in the early years of the survey and in the last two years. Regarding the genus, *Microcystis*, *M. aeruginosa* presented several flowering episodes with high density values.

With regard to the Is Barrocus reservoir (Figure 4), the long-term dynamic shows that the most abundant class over the period of 1996–2002 was that of Cyanobacteria with a peak in the year of 2009 equal to 64.749 (×10^3^ cell/L) composed by *Aphanocapsa* sp. and *Anathece* sp. at 93%. Furthermore, *Aphanocapsa* and *Anathece* gave a strong contribution in terms of density over the entire period, while within the Nostocales, the *Dolichospermum* with the species *D. flos-aquae* showed a significant density over the period of 1996–2004 and regressed in the subsequent years, almost tending to disappear. Another Nostocales that significantly contributed to the composition over the period of 1999–2002, is represented by *Anabaena aphanizomenoides*. *Microcystis* with *M. aeruginosa* showed a similar trend with some blooms until 2006, while disappearing in the following years.

**Figure 7:**
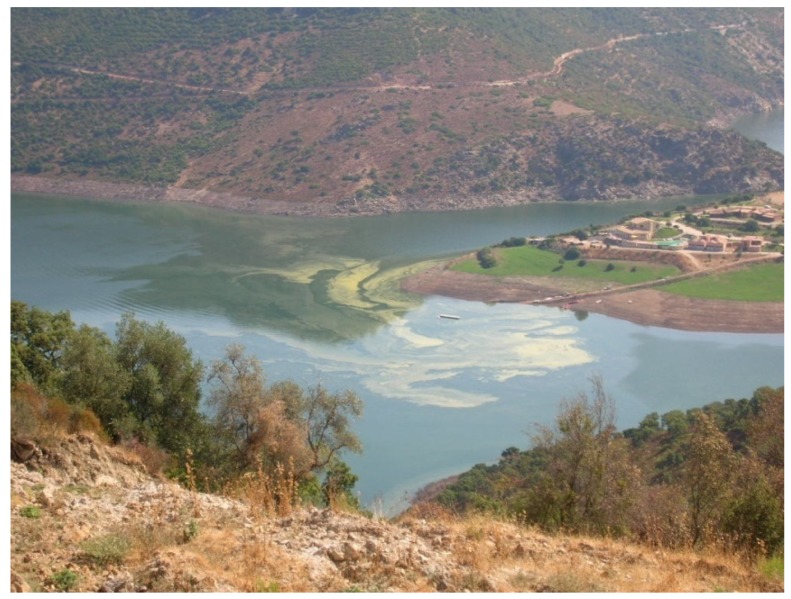
Bloom of *Microcystis* spp. in Flumendosa reservoir.

In Flumendosa reservoir (Figure 5), the dominance was of the Bacillariophyceae and Chlorophyceae classes during the early investigated period. Within the class of Chlorophyceae, the species, *Chlorella sp.* and *Coelastrum reticulatum*, show the highest average density from 1996 to 1999. We observed a massive dominance of the class of Cyanobacteria from 2000 to 2012, with a significant increase in terms of the average density, especially in the last two years. The Cyanobacteria with the highest density values was the *Planktothrix agardhii-rubescens* group, which was very abundant in the years of 2000–2004, with a peak in 2002 of 35.230 (×10^3^ cell/L) corresponding to 99% of the total density class. *Planktothrix agardhii-rubescens* has been tested over the years for their potential toxicity. Furthermore, this species has been responsible for frequent and abundant flowering events and showed an increase in the density values from 2001 to 2012. The *Microcystis* genus was responsible for the onset of blooms in the reservoir (Figure 7), even if with a lower impact than the *Planktothrix* genus. In the species composition, the *Dolichospermum* genus significantly disappeared. Only one bloom occurred in 2003, and sporadically low density values were recorded in the subsequent years.

**Figure 8:**
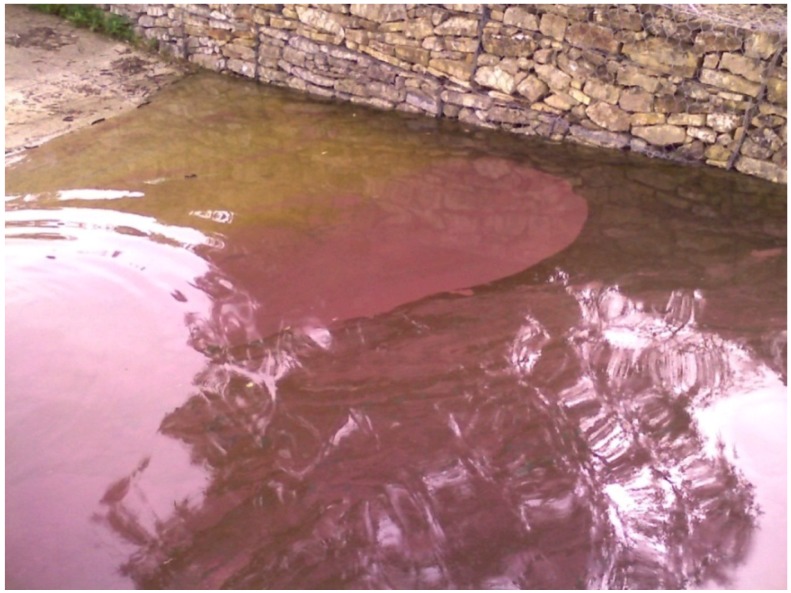
Bloom of *Planktothrix agardhii-rubescens* group in Mulargia reservoir.

A dominance of Chlorophyceae with *Coelastrum reticulatum* and *Pediastrum simplex* was noted in the Mulargia reservoir from 1996 to 1999 (Figure 6) with a peak density of 2.618 (×10^3^ cell/L) in 1999. In the long-term dynamic of algal classes from 2000 to 2012, there is a clear dominance of Cyanobacteria and a good correspondence with the species dynamic. The Cyanobacteria species with the highest occurrence was of the *Planktothrix agardhii-rubescens* group, which became more and more frequent over the years with the occurrence of algal blooms (Figure 8) and which presented a peak density in 2002 with 16.7960 (×10^3^ cell/L) with a dominance of 99%. These species were observed until 2012. Another species of Cyanobacteria contributing significantly to the density of the entire class was *Microcystis* sp. with an important bloom in 2001 recording a density of 115.390 (×10^3^ cell/L) with a dominance of 80%.

Data processed up to this point indicate that in these four reservoirs, by far the most dominant class in the period under consideration was that of Cyanobacteria, responsible for extensive and regular blooms in the system’s reservoirs. Furthermore, the Chlorophyceae, mainly with *Chlorella*, *Coelastrum*, *Oocystis*, *Pediastrum* and *Scenedesmus*, showed significant density values. The third class is represented by Bacillariophyceae with *Cyclotella*, *Aulacoseira*, *Fragilaria* and *Asterionella*, the most frequently found. During algal bloom episodes, their Cyanobacteria concentration can be as high as several million cells per liter, thus creating a significant restraint on the use of the water resource. If we add to this the fact that all three genera (*Planktothrix*, *Aphanizomenon* and *Microcystis*) are known as being toxin-producing, we can easily see how important it is to monitor the quality of water bodies, also from the viewpoint of their phytoplankton composition.

Table 5 shows the results of the algal toxicity test through the water column (0–20 m) carried out in Mulargia and Flumendosa reservoirs during four events of *Planktothrix agardhii-rubescens* blooms in the period of 2010–2012. The results of immunoassays have highlighted the presence of toxic strains of microcystins in both reservoirs. Recently, the “*agardhii-rubescens* group” has received greater attention as being responsible for frequent toxic blooms. The table reports the mean density values of the water column of *P. agardhii-rubescens*, the corresponding date, the intracellular and extracellular concentrations of the microcystins produced, as well as their total value. In fact, the toxins were extracted following the procedure described by Meriluoto and Eriksson [35], which allowed one to separate the two fractions, intracellular and extracellular, which were subsequently treated and assayed with an enzyme immunoassay ELISA. Results show that the highest levels of toxins in Flumendosa were found inside the cells of *Planktothrix agardhii-rubescens* (1.35 µg/L) with a maximum value in dissolved form equal to 0.37 at 2.5 m in depth during the spring of 2011. In Mulargia, the species presented the highest values of toxins inside the cells, with values almost always >2 µg/L and reaching values >5 µg/L at 10 m in depth, those maximum values exceeding the range (0–5 ppb) of the enzyme immunoassay test (ELISA microtiter plate microcystins-ADDA) validity. The extracellular concentrations of microcystins were always lower than 1 µg/L, which is the threshold value for total microcystin-LR (free plus cell-bound) in drinking water established by the World Health Organization (WHO) guideline [36].

**Table 5:** Concentrations of microcystins produced by *Planktothrix agardhii-rubescens* in Flumendosa and Mulargia reservoirs at different depths from four sampling dates.

Reservoirs	Date	Depth (m)	Density (×10^6^ cells L^−1^)	MCYSTs (in)* (µg/L)	MCYSTs (ex)** (µg/L)	MCYSTs (tot)*** (µg/L)
Flumendosa	March 2011	1.0	85	1.3	0.4	1.6
		2.5	84	1.4	0.4	1.5
		5.0	76	1.1	0.2	1.3
		10.0	119	1.0	0.2	1.2
Mulargia	March 2010	0.0	55	2.9	0.0	2.9
		1.0	59	2.7	0.0	2.7
		2.5	59	2.0	0.0	2.0
		5.0	40	1.6	0.0	1.6
		7.5	25	2.5	0.0	2.5
		10.0	32	2.1	0.0	2.1
		15.0	26	1.7	0.0	1.7
		20.0	22	2.3	0.0	2.3
	March 2012	2.5	44	1.3	0.3	1.6
		7.5	48	1.3	0.3	1.6
		10.0	52	>5.0	0.4	>5.0
		15.0	34	1.6	0.3	1.9
	May 2012	7.5	42	2.7	0.2	3.0
		10.0	105	>5.0	0.4	>5.0
		15.0	81	1.7	0.3	2.0
		20.0	76	1.2	0.3	1.4

* MCYSTs (in) = Intracellular microcystins concentration; ** MCYSTs (ex) = Extracellular microcystins concentration; and ***MCYSTs (tot) = Total microcystins concentration.

## 5. Quality Characterization in Management Optimization Modeling

To provide a preliminary evaluation of water system management performances, it is necessary to consider various synthetic quantity and quality datasets that define different hydrological and demand scenarios and related management rules to be optimized. This approach has been partially performed using the WARGI-DSS; here, a more extended time horizon than the historical dataset of Sulis *et al.* [2] is presented to define a comprehensive predictive modeling approach.

A higher level of uncertainty is to be expected in system forecasting using a synthetic quality index dataset (scenario) in the WARGI-DSS optimization of system management. Dealing with synthetic scenarios, several types of relationships have been considered in the regression analysis with the aim of modeling the dependency of *TSI* from other data characterizing reservoirs. Indeed, in order to determine the regression equation, a simple linear least-squares curve was determined between hydrological parameters and *TSI* values at each reservoir. More complicated approaches do not produce significantly better results, at least for the reservoirs under consideration. Therefore, the equation here, used to model the dependence of stored water *TSI*s on stored volumes in a reservoir, is a simple multiple linear regression that incorporates month-explanatory *TSI* variation to allow the curve’s slope to change depending on the month of the year.

As stated above, monthly *TSI* values in the period between 1996 and 2012, were used to calibrate the regression model in the following form:

*TSI_t_* = *a*∙*TSI*_(*t*−1)_ + *b*∙*V_t_* + *c*∙*T* + *d* (9)

where:

*TSL_t_*  evaluated variable at current period *t*;
*TSL_(t−1)_*  evaluated variable at the previous time period;
*T*  transformed time date;
*V_t_*  stored volume in the reservoir at current period *t*;
*a*,*b*,*c*,*d*  regression coefficients.

The number of data pairs, correlation coefficient *R* and residual standard error are listed in Table 6.

**Table 6:** Regression results for *TSI* evaluation in Flumendosa, Mulargia, Is Barrocus and Cixerri reservoirs.

Parameter	Flumendosa	Mulargia	Is Barrocus	Cixerri
Coefficient *R*	0.52	0.56	0.59	0.66
Standard Error	4.25	3.92	3.71	4.22
Number of samplings	145	186	197	242

A large number of chemical, physical and biological factors and their interactions determine the Cyanobacteria density in a reservoir [37,38]. It is widely accepted that TP and TN are the most important factors enhancing the growth of Cyanobacteria *in situ*. In general, nutrient concentrations influence the growth of Cyanobacteria more than the TN:TP ratio. Several observations do not support the significance of the TN:TP ratio [39,40]. The phosphorus-Chl-a models of Dillon and Rigler [14] and Vollenweider [15], already used to implicate phosphorus as an important nutrient in phytoplankton biomass, have also been useful for predicting the concentration of Cyanobacteria or microcystin in reservoirs. Using regression analysis, the best fits for both Chl-a and Cyanobacteria suggest that similar patterns of change in Chl-a and Cyanobacteria occur with increasing TP concentration [41].

Preliminary analyses have been made to verify the interaction over time between Chl-a and Cyanobacteria in the main reservoirs of the Flumendosa-Campidano system and to evaluate the possibility of forecasting Cyanobacteria density. As stated above, monthly *TSI* values in the period between 1996 and 2005, were used to calibrate the regression model in the following form:

ln[*D*(*cyano*)*_t_*] = *a*∙ln[*D*(*cyano*)*_t_*_−1_] + *b*∙*V_t_* + *c*∙*T* + *d* (10)

where:

*D*(*cyano*)_*t*_ evaluated variable at current period *t*;
*D*(*cyano*)_*t*−1_ evaluated variable at the previous time period;
*T*  transformed time date;
*T_t_*  stored volume in the reservoir at current period *t*;
*a*,*b*,*c*,*d*  regression coefficients.

The number of data pairs, correlation coefficient *R* and residual standard error are listed in Table 7.

**Table 7:** Regression results for Cyanobacteria density evaluation in Flumendosa, Mulargia, Is Barrocus and Cixerri reservoirs.

Parameter	Flumendosa	Mulargia	Is Barrocus	Cixerri
Coefficient *R*	0.48	0.62	0.43	0.58
Standard Error	1.32	1.34	1.20	1.52
Number of samplings	144	132	171	178

Following Sulis *et al.* [2], a combined approach evaluates the *QE* index in a reservoir, *j*, at time *t*, using both *TSI* and *D*(*cyano*):

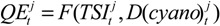
(11)

Considering the threshold of 100 × 10^6^ cell/L, in the case of lower *D*(*cyano*), the *QE* index is based only on *TSI* as reported in Table 1; otherwise, the *QE* value is assumed to equal five (water unsafe for human consumption) no matter the *TSI* value. Based on the value of the state parameters of the water system (stored volume *V* in each reservoir, *j*, at time *t*) under a synthetic hydrological scenario able to reproduce historical drought periods [42], Equations (9)–(11) give the time series of *QE* values to be inserted in the WARGI-DSS in a predictive screening-level model. This integrated approach can provide a preliminary estimation of the impact of different planning alternatives on some quantity and quality issues at the water system scale. Furthermore, it provides an estimate of how the uncertainty of quality data and parameter values can affect different water uses.

## 6. Conclusions

The definition of minimum quality requirements with regard to possible uses is an essential element in the management of complex, multi-user and multi-resource water supply systems, since it limits the actual availability of the resource for use. This paper presents an approach to define a *QE* index to be inserted in a mathematical optimization model for complex water system management. Details on using the water quality index in optimization modeling have been presented in Sulis *et al.* [2]. In this paper, a more extended database is considered to properly define interactions between the most important parameters related to trophic status of lakes and reservoirs, and the relationships from the literature are validated in the Flumendosa-Campidano water system. With regards to this system, the paper presents log-linear relationships between Chl-a, TP and SD to define new *TSI* equations. Furthermore, interactions over time between Chl-a, Cyanobacteria and stored volume were analyzed to evaluate the possibility of forecasting Chl-a and Cyanobacteria density. Further efforts will include the validation of the proposed equation to a larger number of reservoirs in the Island of Sardinia. The analysis shows that the Cyanobacteria was the dominant phytoplankton class in the four reservoirs, and the *Planktothrix* genus was the most abundant in three of them. The results highlight the necessity of paying greater attention to these toxic species in the IWRM of lakes and reservoirs affected by these flowering events. Since the proposed approach is a semi-quantitative immunoassay, a more in-depth and analytical approach could be of value in order to link flowering events with the toxic nature of the responsible species.
